# An immunoinformatics approach for the design of a multi-epitope vaccine targeting super antigen TSST-1 of *Staphylococcus aureus*

**DOI:** 10.1186/s43141-021-00160-z

**Published:** 2021-05-11

**Authors:** Harish Babu Kolla, Chakradhar Tirumalasetty, Krupanidhi Sreerama, Vijaya Sai Ayyagari

**Affiliations:** Department of Biotechnology, Vignan’s Foundation for Science, Technology and Research (Deemed to be University), Vadlamudi, Guntur - District, Andhra Pradesh 522 213 India

**Keywords:** *Staphylococcus aureus*, TSST-1 (Toxic shock syndrome toxin-1), Immunoinformatics, Multi-epitope vaccine

## Abstract

**Background:**

TSST-1 is a secretory and pyrogenic superantigen that is being responsible for staphylococcal mediated food poisoning and associated clinical manifestations. It is one of the main targets for the construction of vaccine candidates against *Staphylococcus aureus*. Most of the vaccines have met failure due to adverse reactions and toxicity reported during late clinical studies. To overcome this, an immunoinformatics approach is being used in the present study for the design of a multi-epitope vaccine to circumvent the problems related to toxicity and allergenicity.

**Results:**

In this study, a multi-epitope vaccine against *Staphylococcus aureus* targeting TSST-1 was designed through an immunoinformatics approach. B cell and T cell epitopes were predicted *in silico* and mapped with linkers to avoid junctional immunogenicity and to ensure the efficient presentation of exposed epitopes through HLA. β-defensin and PADRE were adjusted at the N-terminal end of the final vaccine as adjuvants. Physiochemical parameters, antigenicity, and allergenicity of the vaccine construct were determined with the help of online servers. The three-dimensional structure of the vaccine protein was predicted and validated with various tools. The affinity of the vaccine with TLR-3 was studied through molecular docking studies and the interactions of two proteins were visualized using LigPlot^+^. The vaccine was successfully cloned *in silico* into pET-28a (+) for efficient expression in *E. coli* K12 system. Population coverage analysis had shown that the vaccine construct can cover 83.15% of the global population. Immune simulation studies showed an increase in the antibody levels, IL-2, IFN-γ, TGF-β, B cell, and T cell populations and induced primary, secondary, and tertiary immune responses.

**Conclusion:**

Multi-epitope vaccine designed through a computational approach is a non-allergic and non-toxic antigen. Preliminary *in silico* reports have shown that this vaccine could elicit both B cell and T cell responses in the host as desired.

## Background

*Staphylococcus aureus*, a Gram-positive pathogenic bacterium is responsible for severe clinical obligations. It is a leading cause of several health issues such as food poisoning, skin infections (pus forming), staphylococcal pneumonia, toxic shock syndrome, endocarditis, and osteomyelitis [1–3]. *S. aureus* is a highly versatile pathogen and colonizes axillae, nares, skin, and soft tissues of the body. It is a predominant pathogen causing nosocomial-associated illness among patients with ventilator-associated pneumonia, neutropenia, and among patients who have undergone organ transplantation surgeries. Toxic shock syndrome toxin-1 (TSST-1), a secretory superantigen assists the colonization of *S. aureus* [4, 5]. TSST-1 of *S. aureus* binds to major histocompatibility complex II (MHC II) at a different site than the normal antigens do. It is pyrogenic and is responsible for staphylococcal-mediated food poisoning. It inflates lethal shock by inducing inflammatory cytokines such as Interleukin-1 and tumor necrosis factor [6]. These toxins (superantigens such as TSST-1) are resistant to temperature and gastric conditions like the proteolytic effect of digestive enzymes and extreme acidic pH in the stomach [7]. Staphylococcal food poisoning is associated with vomiting, diarrhea, abdominal cramps, and high fever. Treating staphylococcal infections became very complicated due to the emergence of antibiotic-resistant strains. The bacterium is showing strong resistance to several broad-spectrum antibiotics like Penicillin, Gentamycin, Quinolone, and Erythromycin [8]. The bacterium adapted itself to defend against the antibiotic attack by acquiring antibiotic resistance genes that code for antibiotic hydrolyzing enzymes. With the failure of initial antibiotics, Methicillin was introduced during the early times of the 1960s to treat this pathogen. Unfortunately, Methicillin-resistant *S. aureus* (MRSA) strains have evolved rapidly. Clones of hospital-associated MRSA (HA MRSA) are documented as the leading mortality causative due to nosocomial infections [9–12]. Community-associated MRSA (CA MRSA) infections target healthy individuals. Currently, Vancomycin is used to treat staphylococcal infections as the first line of treatment for HA and CA MRSA infections. Being highly adaptable, *S. aureus* evolved as vancomycin-intermediate (VISA) and Vancomycin-resistant (VRSA) with increased virulence [13–15]. Many vaccine models have been developed against *S. aureus*. But a majority of the vaccines are in the development and evaluation stages of preclinical and clinical trials. Some of the models remained a failure in the late stages of clinical trials due to multiple factors like severe toxicity and extreme allergic reactions [3, 16]. Hence, immunoinformatics tools with reverse vaccinology principles are being used currently for the design of multi-epitope vaccines to minimize the time of vaccine discovery and reduce the toxicity and allergic reactions. Multi-epitope vaccines include B cell and T cell epitopes mapped together, thereby maximizing the efficacy with sharp immunogenicity.

In the present study, we designed a multi-epitope vaccine and evaluated its physicochemical attributes targeting TSST-1 protein to confer protection against staphylococcal infections. A brief account of the various immunoinformatics tools used in the present study is given below.

Online resources and tools such as BepiPred-2.0, NetCTLpan 1.1, and NetMHCIIpan 4.0 were used in the present study to predict the epitopes in the TSST-1. BepiPred-2.0 is an online tool that uses a random forest algorithm that was trained on epitopes derived from antigen–antibody structures to predict the epitopes in a given protein sequence [17]. NetCTLpan 1.1 is used to predict the epitopes that bind to MHC-I molecules. NetCTLpan 1.1 relies on the predictions of (i) proteasomal C-terminal cleavage (Cle), (ii) binding affinities of the epitopes to MHC I complex (MHC), and (iii) TAP transport efficiency (TAP). The overall prediction scores (Comb) are derived as a weighted average of the above three parameters. The method has been validated and trained on experimentally derived large data sets consisting of CTL epitopes and MHC Class I ligands [18]. NetMHCIIpan-4.0 employs an algorithm termed NNAlign_MA for the analysis and integration of the large-scale mass spectrometry data obtained for the peptides that were eluted from MHC-II complexes for the prediction of CD4^+^ epitopes [19]. ‘IFNepitope’ is the first online tool developed to predict IFN-γ-inducing peptides. The algorithm relies on a motif-based, support vector machine (SVM)-based and hybrid (motif and SVM) approaches for predicting IFN-γ epitopes [20]. The Immune Epitope Database (IEDB) comprises allelic and epitope information. This resource was used to study population coverage analysis of the vaccine construct. The database comprises sets of alleles corresponding to the different population groups of the world [21]. The vaccine model was evaluated for its immunological properties such as antigenicity, allergenicity, and toxicity by employing VaxiJen 2.0, AllerTOP 2.0, and ToxinPred servers respectively. Vaxijen 2.0 is the first alignment-independent tool developed to determine the antigenicity of a given peptide. The tool purely relies on the physicochemical properties of the peptide rather than alignment [22]. AllerTOP v. 2.0 is based on auto cross-covariance (ACC) transformation of protein sequences into uniform equal-length vectors [23]. ToxinPred predicts the toxicity of the peptide-based on four parameters like amino acid composition, dipeptide composition, binary profile pattern, and motif-based profile [24]. Physicochemical parameters of the vaccine protein were evaluated with the help of the Protparam tool available at ExPasy. The program generates output parameters like the grand average of hydropathicity (GRAVY), theoretical pI, amino acid composition, aliphatic index, extinction coefficient, atomic composition, etc. [25]. The amino acid sequence was reverse translated to the corresponding nucleotide sequence through the codon adaptation tool—JCat [26]. It works on the Java environment to convert peptide sequence to nucleotide sequence. Immune response in the mammalian system for the vaccine was simulated using the C-IMMSIM server. The server simulates the mammalian immune system and generates antibody levels, antigen clearance from the system, T cell and B cell population, etc. [27]. Structural properties of the vaccine were evaluated through molecular modeling, structure refinement, and molecular docking, among others. SnapGene v5.1.4.1 was used for cloning the vaccine gene into a pET28a (+) vector for expression in a prokaryotic system.

## Methods

**Fig. 1:**
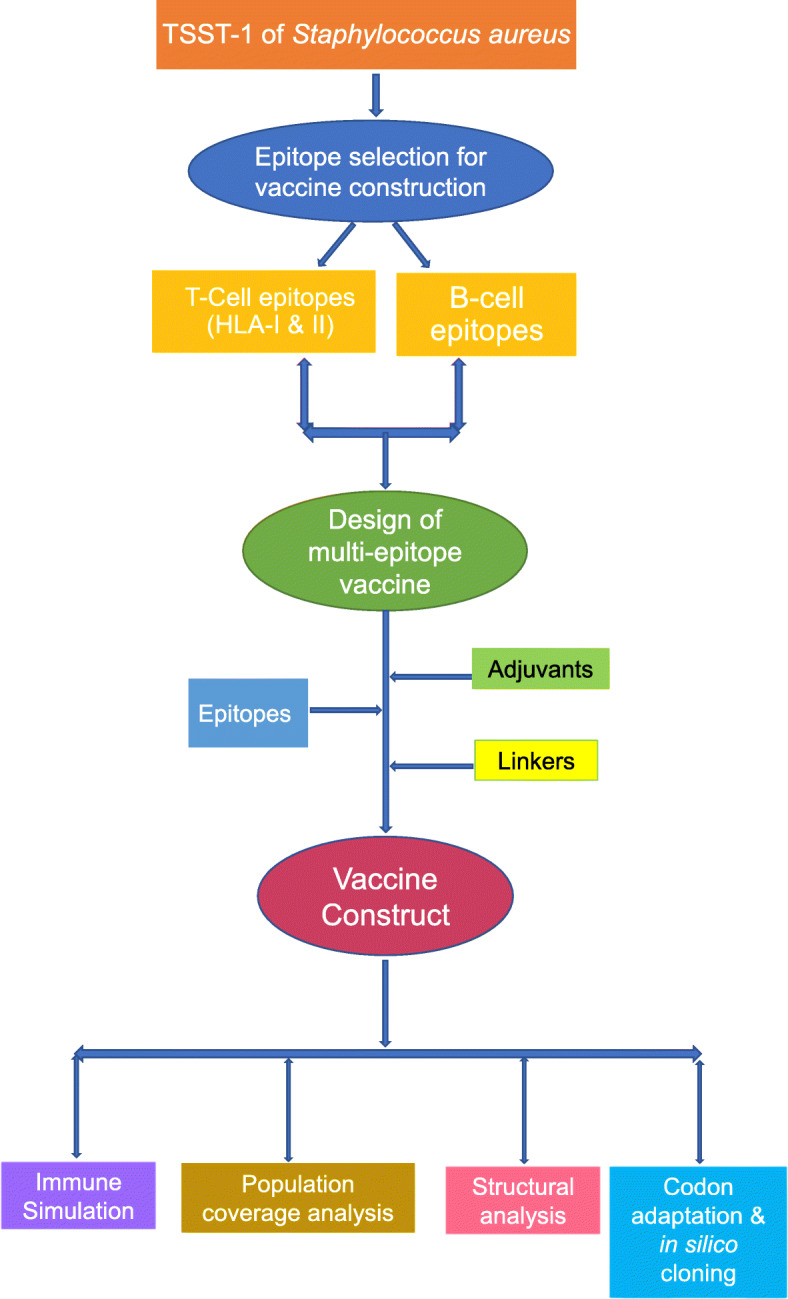
Flow chart representing the schematic view of *in silico* design of multi epitope vaccine

The flow chart in Fig. 1 describes the process of multi-epitope vaccine design targeting the TSST-1 protein of *Staphylococcus aureus*.

### Sequence retrieval

Two hundred thirty-four-AA long peptide sequence of TSST-1 protein with ID P06886 of *Staphylococcus aureus* was retrieved from Uniprot database (*https://www.uniprot.org/uniprot/P06886*). Antigenicity and allergenicity of the native protein were determined with VaxiJen (*http://www.ddg-pharmfac.net/vaxijen/VaxiJen/VaxiJen.html*) [22] and AllerTOP 2.0 (*https://www.ddg-pharmfac.net/AllerTOP/*) servers [23].

### B cell epitope prediction

BepiPred-2.0 (*http://www.cbs.dtu.dk/services/BepiPred/*) [17] server was used for predicting the B cell epitopes in the protein. Epitopes greater than or equal to 10-AA long at the default threshold of 0.5 were only considered for downstream analyses.

### HLA-I & -II alleles

Twenty seven HLA-I and 27 HLA-II alleles (Table 1) that occur most frequently in the global population were considered for T cell epitope prediction (*https://help.iedb.org/hc/en-us/articles/114094151851* accessed on 05 July 2020 and the appropriate references cited therein) [21].

**Table 1:** Final HLA-I epitopes for vaccine construction

Epitope	Allele	Antigenicity	Allergenicity	Toxicity
ATDFTPVPL	HLA-A*01:01	Antigen (0.7520)	Non-allergen	Non-toxin
LIIFPSPYY	HLA-A*01:01	Antigen (1.3917)	Non-allergen	Non-toxin
RSSDKTGGY	HLA-A*01:01	Antigen (2.2165)	Non-allergen	Non-toxin
KLLMNFFIV	HLA-A*01:01	Antigen (2.0829)	Non-allergen	Non-toxin
TTATDFTPV	HLA-A*02:03	Antigen (0.7862)	Non-allergen	Non-toxin
KLLMNFFIV	HLA-A*02:06	Antigen (2.0829)	Non-allergen	Non-toxin
TTATDFTPV	HLA-A*02:06	Antigen (0.7862)	Non-allergen	Non-toxin
LIIFPSPYY	HLA-A*03:01	Antigen (1.3917)	Non-allergen	Non-toxin
STYQSDLSK	HLA-A*03:01	Antigen (0.5158)	Non-allergen	Non-toxin
STYQSDLSK	HLA-A*11:01	Antigen (0.5158)	Non-allergen	Non-toxin
FFIVSPLLL	HLA-A*23:01	Antigen (2.0269)	Non-allergen	Non-toxin
WYSSGSDTF	HLA-A*23:01	Antigen (0.4769)	Non-allergen	Non-toxin
FFIVSPLLL	HLA-A*24:02	Antigen (2.0269)	Non-allergen	Non-toxin
WYSSGSDTF	HLA-A*24:02	Antigen (0.4769)	Non-allergen	Non-toxin
TTATDFTPV	HLA-A*26:01	Antigen (0.7862)	Non-allergen	Non-toxin
LIIFPSPYY	HLA-A*26:01	Antigen (1.3917)	Non-allergen	Non-toxin
LIIFPSPYY	HLAA*30:02	Antigen (1.3917)	Non-allergen	Non-toxin
HGKDSPLKY	HLAA*30:02	Antigen (1.2582)	Non-allergen	Non-toxin
TTATDFTPV	HLA-A*68:02	Antigen (0.7862)	Non-allergen	Non-toxin
FPSPYYSPA	HLA-B*07:02	Antigen (0.4330)	Non-allergen	Non-toxin
LPTPIELPL	HLA-B*07:02	Antigen (0.5984)	Non-allergen	Non-toxin
SPLKYGPKF	HLA-B*07:02	Antigen (0.9748)	Non-allergen	Non-toxin
LIIFPSPYY	HLA-B*15:01	Antigen (1.3917)	Non-allergen	Non-toxin
QLAISTLDF	HLA-B*15:01	Antigen (1.1049)	Non-allergen	Non-toxin
FPSPYYSPA	HLA-B*35:01	Antigen (0.4330)	Non-allergen	Non-toxin
LPTPIELPL	HLA-B*35:01	Antigen (0.5984)	Non-allergen	Non-toxin
SPLKYGPKF	HLA-B*35:01	Antigen (0.9748)	Non-allergen	Non-toxin
FPSPYYSPA	HLA-B*51:01	Antigen (0.4330)	Non-allergen	Non-toxin
LPTPIELPL	HLA-B*51:01	Antigen (0.5984)	Non-allergen	Non-toxin
LPTPIELPL	HLA-B*53:01	Antigen (0.5984)	Non-allergen	Non-toxin
SPLKYGPKF	HLA-B*53:01	Antigen (0.9748)	Non-allergen	Non-toxin

### HLA-I restricted epitopes

HLA-I epitopes of TSST-1 protein were predicted with NetCTLpan 1.1 server (https://services.healthtech.dtu.dk/service.php?NetCTLpan-1.1) [18] at the default threshold value 1.0. The output of the program gives all the possible epitopes with the respective threshold scores. Peptides of threshold value less than or equal to the default value 1.0 were only considered for epitope mapping.

### HLA-II restricted epitopes

NetMHCIIpan–4.0 tool (https://services.healthtech.dtu.dk/service.php?NetMHCIIpan-4.0) was employed for the identification of epitopes associated with HLA-II alleles [19]. This program generates information regarding peptide sequence, core peptide, and binding affinities. Epitopes that have strong affinities (SA) towards HLA-II were only selected for vaccine design. Default threshold values corresponding to the strong and weak affinities between epitopes and MHC II molecules are 1.0 and 0.5, respectively.

### Epitope mapping and vaccine construction

Selected B and T cell epitopes were coupled with adjuvant sequences with the help of linkers. Adjuvant and PADRE sequences were joined with the EAAAK linker. AAY, GPGPG and KK linkers were used to join CTL, HTL, and B cell epitopes. The vaccine sequence was tagged with hexahistidine at the C-terminal end to aid protein purification by Ni-NTA chromatography. GPGPG, AAY, and KK linkers are the most widely used linkers in epitope mapping for the design of multi-epitope vaccines. Linkers are used to avoid junctional immunogenicity and enhance the sharp immunity towards the pathogen [21]. β-defensin and PADRE sequence were added as adjuvants at the N-terminal end to increase the immunogenicity of the vaccine. β-defensins are anti-microbial peptides involved in innate immunity and have a strong affinity to TLRs (e.g., TLR3) [21]. PADRE sequence was added at the N-terminal end to increase the immunogenicity of the vaccine [21].

### Evaluation of vaccine construct

Immunological and physicochemical properties of the final vaccine construct were determined through *in silico* screening by employing various tools. Antigenicity and allergenicity of the vaccine were determined with VaxiJen (*http://www.ddg-pharmfac.net/vaxijen/VaxiJen/VaxiJen.html*) and AllerTOP 2.0 (*https://www.ddg-pharmfac.net/AllerTOP/*) servers respectively. These two properties are compared between the native TSST-1 protein and *in silico* processed multi-epitope vaccine. The toxicity of the epitopes was predicted with the ToxinPred server (*https://webs.iiitd.edu.in/raghava/toxinpred/design.php*) [24]. Physicochemical properties in terms of molecular weight, amino acid composition, atomic composition, and grand average of hydropathicity (GRAVY) were determined with ProtParam online resource (*https://web.expasy.org/protparam/*) [25].

### Interferon-γ inducing epitope prediction

Interferon-γ (IFN-γ) inducing epitopes of the vaccine construct were determined with the ‘Motif and SVM hybrid’ method in IFNepitope (*https://webs.iiitd.edu.in/raghava/ifnepitope/scan.php*) [20]. This tool generates epitopes of 15 AA in length and predicts the IFN-γ inducing ability of each peptide at threshold 1.

### Population coverage analysis

HLA types and the frequencies of their occurrence vary among the global population, which determine the antigen processing and epitope presentation through HLA-I and -II. To understand the extent of coverage of the global population by the vaccine, the population coverage analysis module in the IEDB resource was used (*http://tools.iedb.org/population/*). Population coverage analysis for both HLA-I & -II was carried out in the study by area, country, and ethnicity.

### Immune simulations

C-IMMSIM (*http://150.146.2.1/C-IMMSIM/index.php?page=1*) [27] (accessed on December 13, 2020) server was used in this study to simulate and understand the nature of the vaccine construct as antigen in eliciting the immune responses in the host. For simulating immune response, HLA-I alleles viz. HLA-A (HLA-A01:01, HLA-A02:01), HLA-B (HLAB15:01, HLA-B57:01), and HLA-II alleles—DRB1_0101 and DRB1_1302 were considered randomly as a heterozygous combination in the host. Three injections of the vaccine construct without LPS were administered at intervals of 4 weeks by following one time step as 8 h. All the parameters (i.e., simulation speed, random speed) were set at their default parameters. Simulation steps were set to 1050 with simulation volume 1 μL.

### Codon adaptation

For codon adaptation of the final vaccine construct in *Escherichia coli* K12 system, JCat server (JAVA Codon adaptation tool) (*http://www.jcat.de/Start.jsp*) was used following the standard genetic code for the *in silico* conversion of input amino acid sequence to DNA sequences [26]. The following parameters viz. rho-independent transcription terminators, prokaryotic ribosome binding sites, and cleavage sites of restriction enzymes were chosen to generate an optimized DNA sequence concerning the input amino acid sequence of the vaccine construct.

### Prediction, validation, and refinement of vaccine structure

The secondary structure of the vaccine construct was predicted through PSIPRED 4.0 available at *http://bioinf.cs.ucl.ac.uk/ psipred/* [28, 29]. The tertiary structure of the vaccine protein was predicted through I-TASSER (Iterative Threading ASSEmbly Refinement) server available at *https://zhanglab.ccmb.med.umich.edu/I-TASSER/* [30]. I-TASSER follows the template-based prediction of protein employing LOMETS threading program. It predicts the top 5 models with a corresponding Confidence score (C-score). The quality of each model was determined by C-score (i.e., a higher C-score represents higher confidence with that model). After the prediction of the tertiary structure of the protein, the protein file with extension .*PDB* was submitted to GalaxyWEB server (GalaxyRefine2) (*http://galaxy.seoklab.org/ index.html*) for its refinement [31]. The refined tertiary structure of the vaccine candidate was further validated with the ProSA-web server (*https://prosa.services.came.sbg.ac.at/prosa.php*) [32, 33]. Z scores of vaccine structure generated by I-TASSER and the refined model were compared to validate the overall structural quality. Ramachandran plot of the vaccine structure was obtained with UCSF Chimera software.

### Molecular docking

Molecular docking between vaccine (ligand) and TLR3 (receptor) was carried out to study the affinity and interaction between the molecules. TLR3 was chosen as a receptor because it is an antagonist to β-defensin in eliciting immune response [21]. β-defensin is included in the final vaccine sequence as an adjuvant to enhance its efficacy. PDB file of TLR3 (PDB ID: 1ZIW) was downloaded from the RCSB PDB server (*https://www.rcsb.org/structure/1ZIW*). Water molecules and native ligands or inhibitors were removed from the structure using PyMOL software. Molecular docking was carried out between ligand (vaccine—Chain B) and receptor (TLR3—Chain A) using PatchDock server (*https://bioinfo3d.cs.tau.ac.il/PatchDock/php.php*) [34]. The top 10 models generated by the PatchDock server were further submitted to the FireDock server (http:// bioinfo3d.cs.tau.ac.il/FireDock/php.php) [35] to yield a refined vaccine-TLR3 complex. Two-dimensional interactions between the receptor and ligand in the docked complex were studied using the DIMPLOT module in LigPlot^+^ v2.2 software.

### *In silico* cloning

SnapGene v5.1.4.1 was used for *in silico* cloning of the gene insert into pET-28a (+) vector for the expression of vaccine protein in the prokaryotic system (*E coli* K12). Prior to cloning, the insert was assessed for the presence of restriction sites in it. After the confirmation for the presence of restriction sites, restriction-specific sequences were tagged at N-terminal and C-terminal ends of the vaccine gene insert for efficient cloning.

## Results

### Prediction of B cell epitopes

BepiPred server predicted a total of 9 epitopes of different lengths in TSST-1 protein (234 amino acids) of *Staphylococcus aureus*. Only those epitopes greater than or equal to 10 amino acids in length were selected for vaccine construction. Antigenicity, toxicity, and allergenicity of these epitopes were predicted, and epitopes that are antigenic but neither allergic nor toxic were finally included in the vaccine. TKKSQHTSEGTY is the only antigenic epitope that is neither allergic nor toxic. Hence, this epitope was included in the final vaccine construct.

### Prediction of T cell epitopes

A total of 69 and 95 HLA-I and -II restricted T cell epitopes were identified through NetCTLpan and NetMHCpan tools, respectively. No epitopes were identified concerning certain HLA alleles viz. HLA-A*30:01, HLA-A*32:01, HLA-A*68:01, HLA-B*08:01, HLA-B*44:02, HLA-B*44:03, HLA-DRB1*01:01, HLA-DRB1*09:01, and HLA-DPA1*03:01/DPB1*04:02. All the final epitopes were again filtered for vaccine design based on their toxicological and immunological aspects. After the prediction of antigenicity, allergenicity, and toxicity, all the filtered epitopes corresponding to their HLA alleles were sorted (Tables 1 and 2). Among the filtered epitopes, repeated epitopes were ignored, and unique epitopes were analyzed for vaccine construction. Besides, a few HLA-I restricted epitopes were overlapping with certain HLA-II restricted epitopes. For instance, HLA-I epitopes FPSPYYSPA, QLAISTLDF, and RSSDKTGGY were overlapping with HLA-II epitopes FPSPYYSPAFTKGEK, DKKQLAISTLDFEIR, and QIHGLYRSSDKTGYY. Such epitopes were rationally filtered by considering one among all the overlapping epitopes.

**Table 2:** Final HLA-II epitopes for vaccine construction

Epitope	Allele	Antigenicity	Allergenicity	Toxicity
SNQIIKTAKASTNDN	HLA-DRB1*08:02	Antigen (0.4273)	Non-allergen	Non-toxic
SNQIIKTAKASTNDN	HLA-DRB1*11:01	Antigen (0.4582)	Non-allergen	Non-toxic
QIHGLYRSSDKTGGY	HLA-DRB1*11:01	Antigen (0.7278)	Non-allergen	Non-toxic
SNQIIKTAKASTNDN	HLA-DRB1*13:02	Antigen (0.4582)	Non-allergen	Non-toxic
MNDGSTYQSDLSKKF	HLA-DRB3 *01:01	Antigen (0.8123)	Non-allergen	Non-toxic
NDGSTYQSDLSKKFE	HLA-DRB3 *01:01	Antigen (1.0840)	Non-allergen	Non-toxic
STYQSDLSKKFEYNT	HLA-DRB3 *01:01	Antigen (0.7472)	Non-allergen	Non-toxic
TYQSDLSKKFEYNTE	HLA-DRB3 *01:01	Antigen (0.8673)	Non-allergen	Non-toxic
SKKFEYNTEKPPINI	HLA-DRB3*02:02	Antigen (0.9356)	Non-allergen	Non-toxic
KKFEYNTEKPPINID	HLA-DRB3*02:02	Antigen (1.0198)	Non-allergen	Non-toxic
KFEYNTEKPPINIDE	HLA-DRB3*02:02	Antigen (1.0332)	Non-allergen	Non-toxic
LGSMRIKNTDGSISL	HLA-DRB4*01:01	Antigen (1.2178)	Non-allergen	Non-toxic
DKKQLAISTLDFEIR	HLA-DRB4*01:01	Antigen (1.4067)	Non-allergen	Non-toxic
SNQIIKTAKASTNDN	HLA-DQA1*05:01/DQB1*03:01	Antigen (0.4582)	Non-allergen	Non-toxic
QIIKTAKASTNDNIK	HLA-DQA1*05:01/DQB1*03:01	Antigen (0.5176)	Non-allergen	Non-toxic

### Vaccine design

**Fig. 2:**
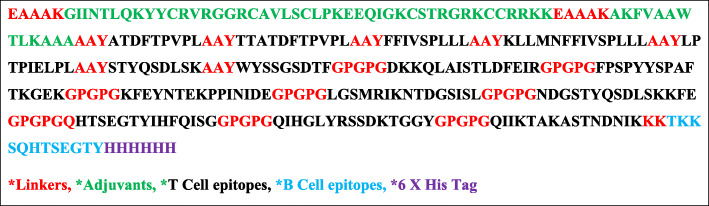
Multi-epitope vaccine constructed by mapping both B cell and T cell epitopes with linkers and adjuvants

The vaccine is 339 amino acids long with one B cell epitope, 7 HLA-I epitopes, and 8 HLA-II epitopes (Fig. 2). β-defensin and PADRE sequence were added as adjuvants at the N-terminal end.

### Evaluation of vaccine construct

The vaccine construct designed in the study was evaluated for its immunological and physiochemical attributes. The native TSST-1 protein of *Staphylococcus aureus* and the *in silico* designed vaccine sequence were compared for their antigenicity and allergenicity. It was observed that native TSST-1 protein is an allergic antigen (probability as protective antigen = 0.8730), whereas the multi-epitope vaccine designed in the present study is a non-allergic antigen (probability as protective antigen = 0.9748).

IFN-γ inducing epitope prediction for vaccine sequence was performed. IFN-γ is crucial for both innate and adaptive immunity [36]. Activated T cells and NK (natural killer) cells secrete IFN-γ for promoting the activation of macrophages and antiviral mechanisms and enhancing the production of antibodies [36]. A total of 85 positive and 261 negative IFN-γ inducing epitopes were identified corresponding to the multi-epitope vaccine designed in the present study.

Population coverage analysis of the vaccine suggests that the multi-epitope vaccine could cover over 83.15% of the global population (HLA-I and -II combined).

The molecular weight and theoretical pI of the vaccine were 36.30 kDa and 9.34, respectively. The vaccine is basic based on the theoretical pI value. The extinction coefficient of the vaccine protein was found to be 41,175 M^-1^ cm^-1^ at 280 nm. The total number of positively charged amino acids (Asp + Glu) in the protein was 25, whereas the total number of negatively charged amino acids (Arg + Lys) was 39. The estimated half-life of the vaccine is 1 h in mammalian reticulocytes (*in vitro*) and 30 min in yeast (*in vivo*) and > 10 h in *Escherichia coli* (*in vivo*). The instability index of the vaccine construct was computed to be 31.93, thus classifying the vaccine as a stable protein. Protein that has an instability index of greater than 40 is unstable (*https://web.expasy.org/protparam/protparam-doc.html* (accessed on December 18, 2020)). The aliphatic index and GRAVY (grand average of hydropathicity) of the protein were 63.75 and − 0.458, respectively.

### Immune simulation

**Fig. 3:**
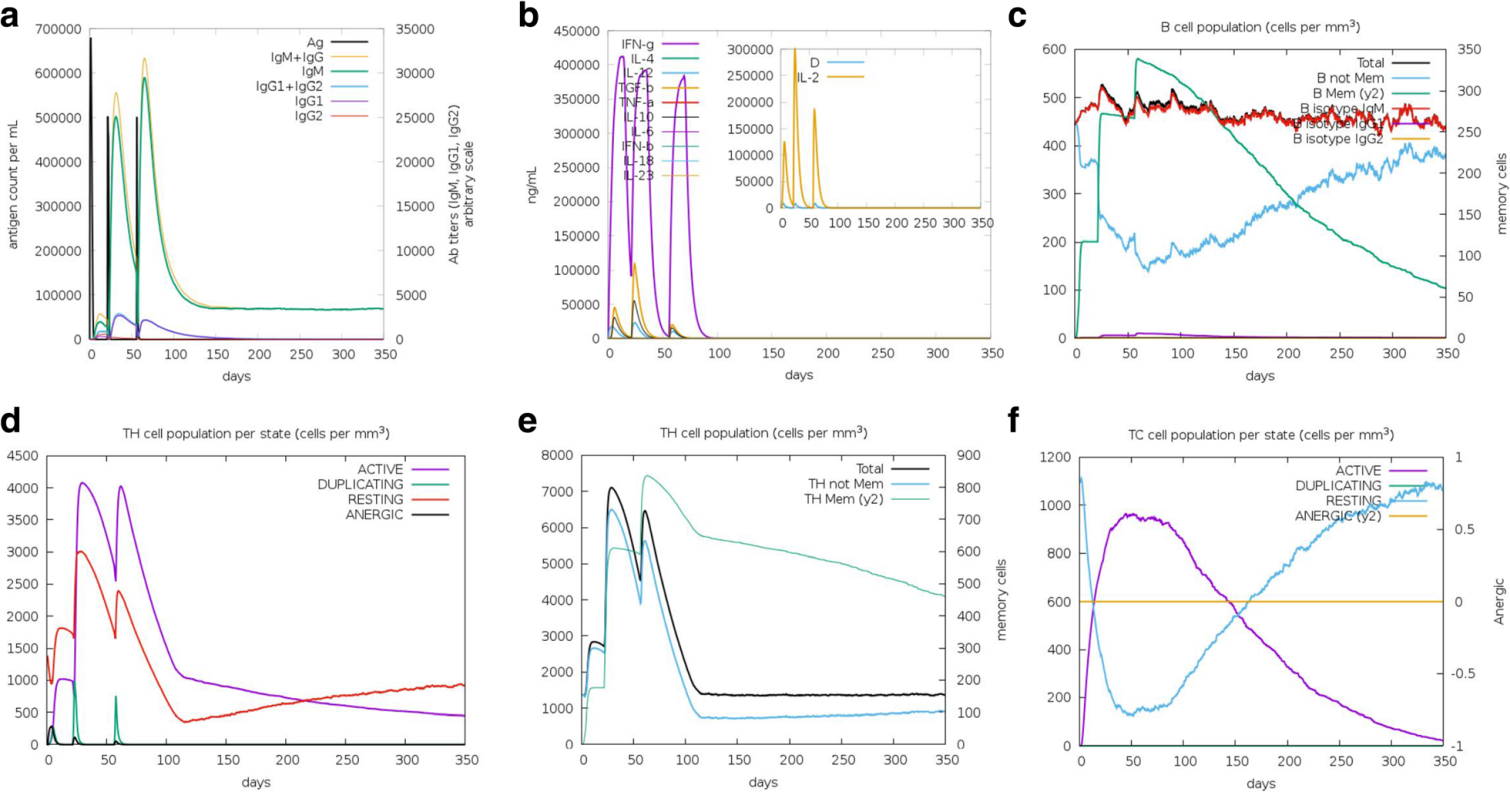
Immune response to the vaccine construct in mammalian system generated through CIMMSIM server. **a** Antigen count (per ml) and antibody levels (IgM, IgG1, and IgG2). **b** Cytokine levels in the system (ng per ml). **c** B cell population (per mm^3^). **d** TH cell population per state (per mm^3^). **e** TH cell population (per mm^3^). **f** TC cell population per state (per mm^3^)

Results obtained from the C-IMMSIM server showed higher levels of IgM, IgM + IgG, IgG1 + IgG2, and IgG1 in secondary and tertiary immune responses. Antigen levels decreased at each level of immune response with the rise in antibody level (Fig. 3a). The decline in antigen levels imputes a rise in total B cell and T cell counts (Fig. 3c, d, e, f). During secondary and tertiary immune responses, active, memory, total T_H_, and T_C_ cells per state were also increased when compared with primary immune responses. Through immune simulation studies, it was clear that the levels of B lymphocytes, T lymphocytes, and antibodies were increased with the administration of antigen thus ensuring the clearance of antigen from the system through primary, secondary, and tertiary immune responses.

### Structure prediction and validation

**Fig. 4:**
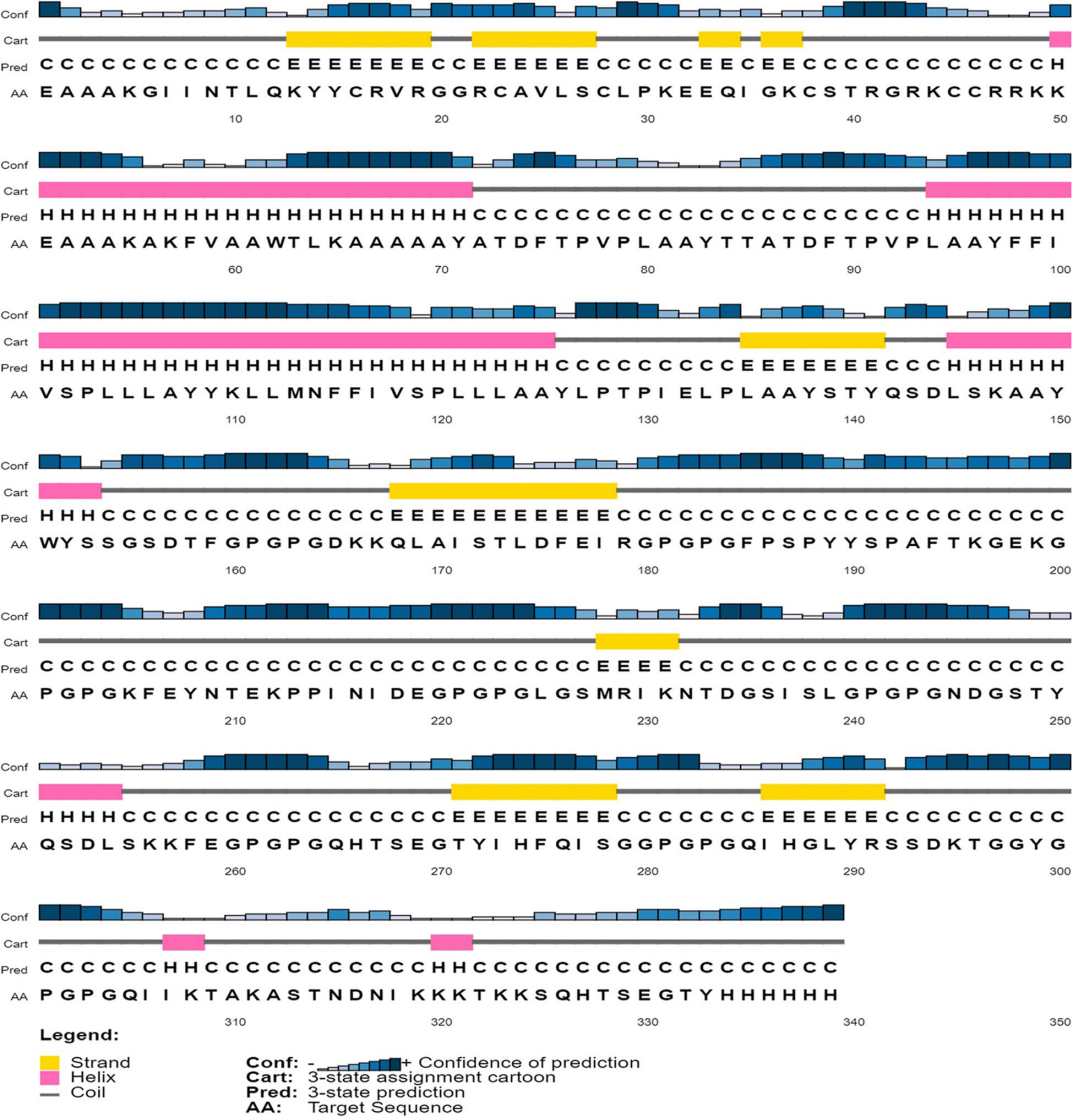
Secondary structure of the vaccine protein constructed through PSIPRED server

The secondary structure of the vaccine protein was predicted with the PSIPRED 4.0 server (*http://bioinf.cs.ucl.ac.uk/psipred/*) [28, 29] (Fig. 4). This program generated secondary structural elements of the protein such as strands, helix, and coil. The majority of the structure has coils (63.4%). Whereas the percentage contents of alpha helix and beta strand are 20% and 15.6%, respectively.

### Tertiary structure prediction and refinement

**Fig. 5:**
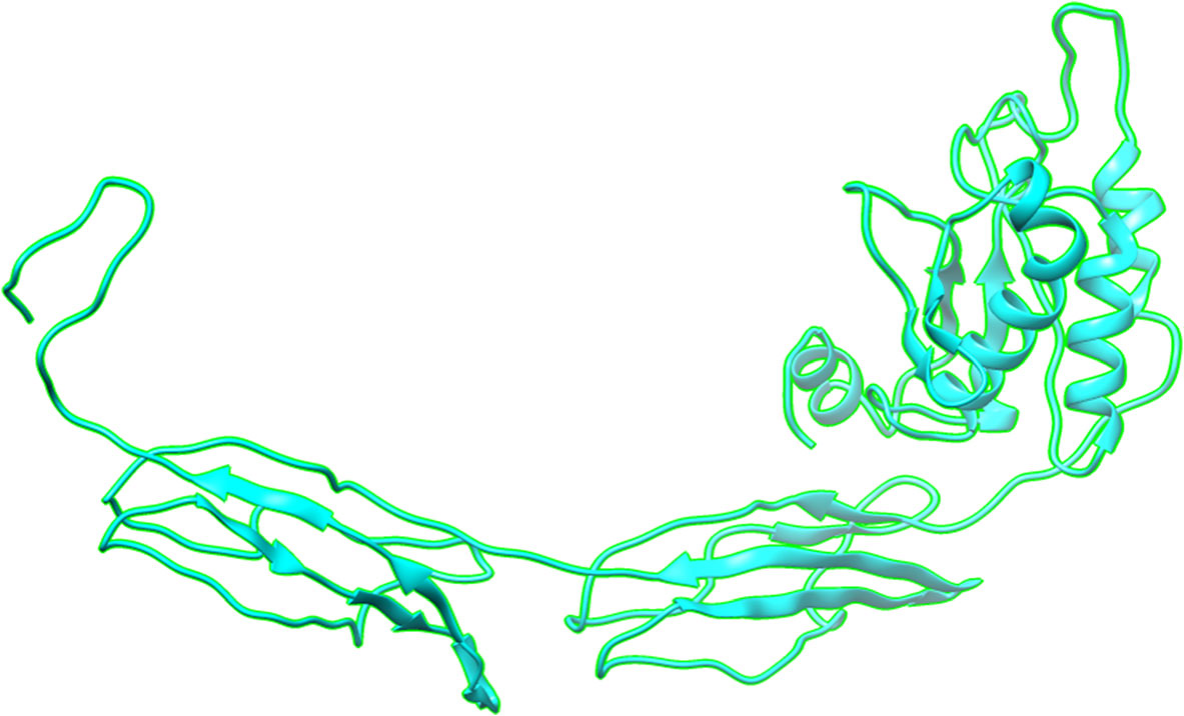
3D Tertiary structure of the vaccine generated by I-TASSER server and further refined with GalaxyWeb

**Fig. 6:**
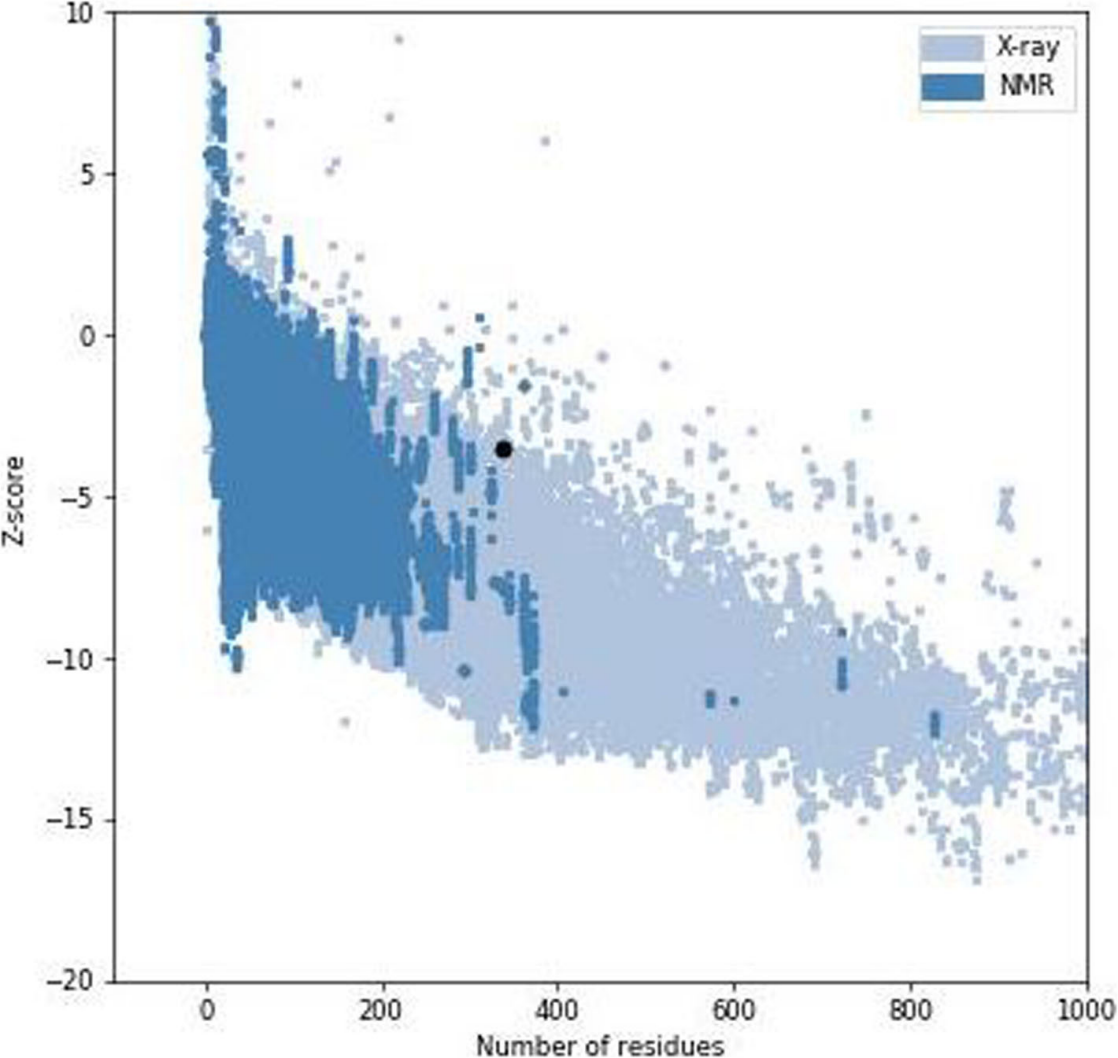
Z score of the model of the vaccine model developed by ProSA-web

The tertiary structure was predicted through the I-TASSER program. The program generated a total of five models. The top model with C-score − 2.49, estimated RMSD 12.4 + 4.3 Å, and estimated TM-score 0.42 + 0.14 was considered as the best model and was utilized for carrying out structural refinement. GalaxyWeb server was used to refine the tertiary structure of the protein. After refinement, model 4 (RMSD 0.408 and MolProbity 2.366) was selected out of 5 models generated by the GalaxyWeb server for further analyses (Fig. 5). The Z score of the model before and after refinement was − 3.21 and − 3.54, respectively (Fig. 6). Ramachandran plot for the refined model generated with UCSF Chimera showed that 97% of the residues were in allowed regions, which is an indication of a good protein structure.

### Molecular docking

**Fig. 7:**
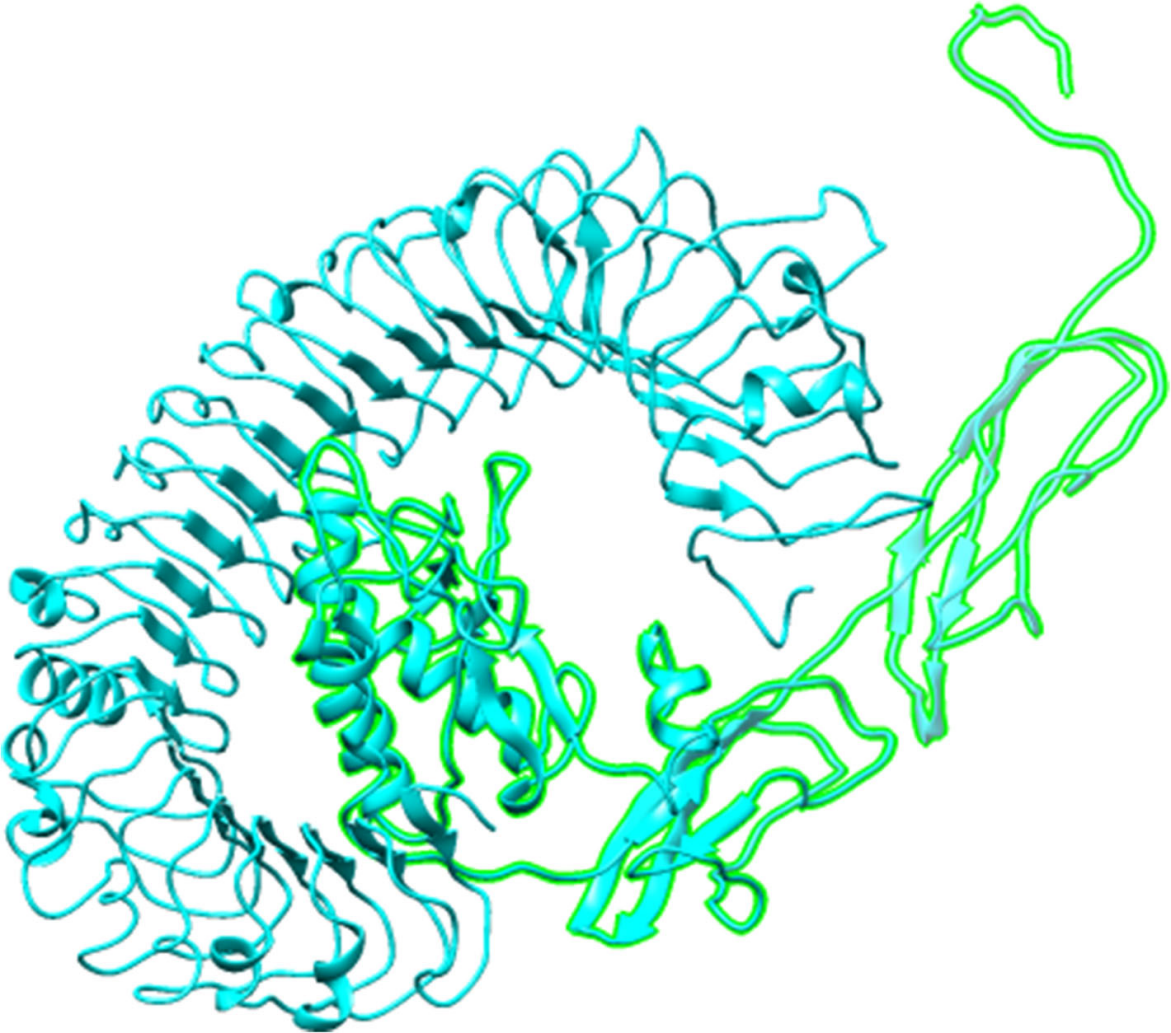
Docked complex of TLR3 (receptor) and vaccine (ligand) generated by PatchDock. TLR3 is represented in Blue color and the Vaccine is represented in Green color

TLR3 (receptor) and vaccine (ligand) were docked using the PatchDock server. Solutions generated from the PatchDock program were further refined using FireDock. Solution 9 (Fig. 7) of all the 10 outputs of PatchDock was demonstrated as the best model by FireDock. This solution was ranked number one with global energy of − 5.83 KCalMol^-1^.

**Fig. 8:**
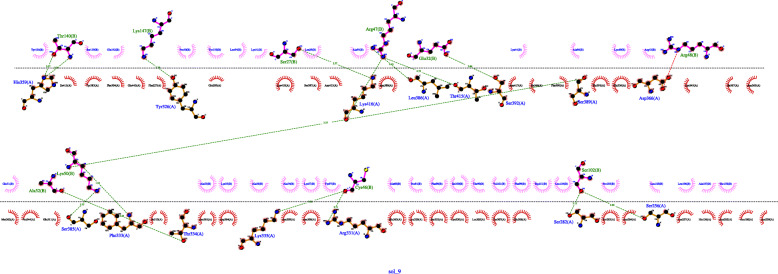
2D interactions of vaccine (Chain B)-TLR3 receptor (Chain A) docked complex visualized through Dimplot module in Ligplot

Two-dimensional interactions between the vaccine and TLR3 (Fig. 8) were visualized with the DIMPLOT module in LigPlot^+^ software.

### Codon adaptation

After mapping B cell and T cell epitopes with linkers and adjuvants, the final vaccine construct consisted of 339 amino acids. The total number of nucleotides corresponding to the protein was identified with the help of JCat. After codon adaptation, the program generated an output of 1017 bp DNA sequence. CAI value and GC content of the improvised sequence were 0.95 and 53.6%, respectively. CAI value of > 0.8 and an optimal GC content of range 30–70% is considered for good expression of the gene in the host [26]. Therefore, both the parameters, i.e. CAI value and GC content, were highly satisfactory for the efficient expression of recombinant multi-epitope TTST-1 in *E coli* K12 cells [37, 38].

### *In silico* cloning

**Fig. 9:**
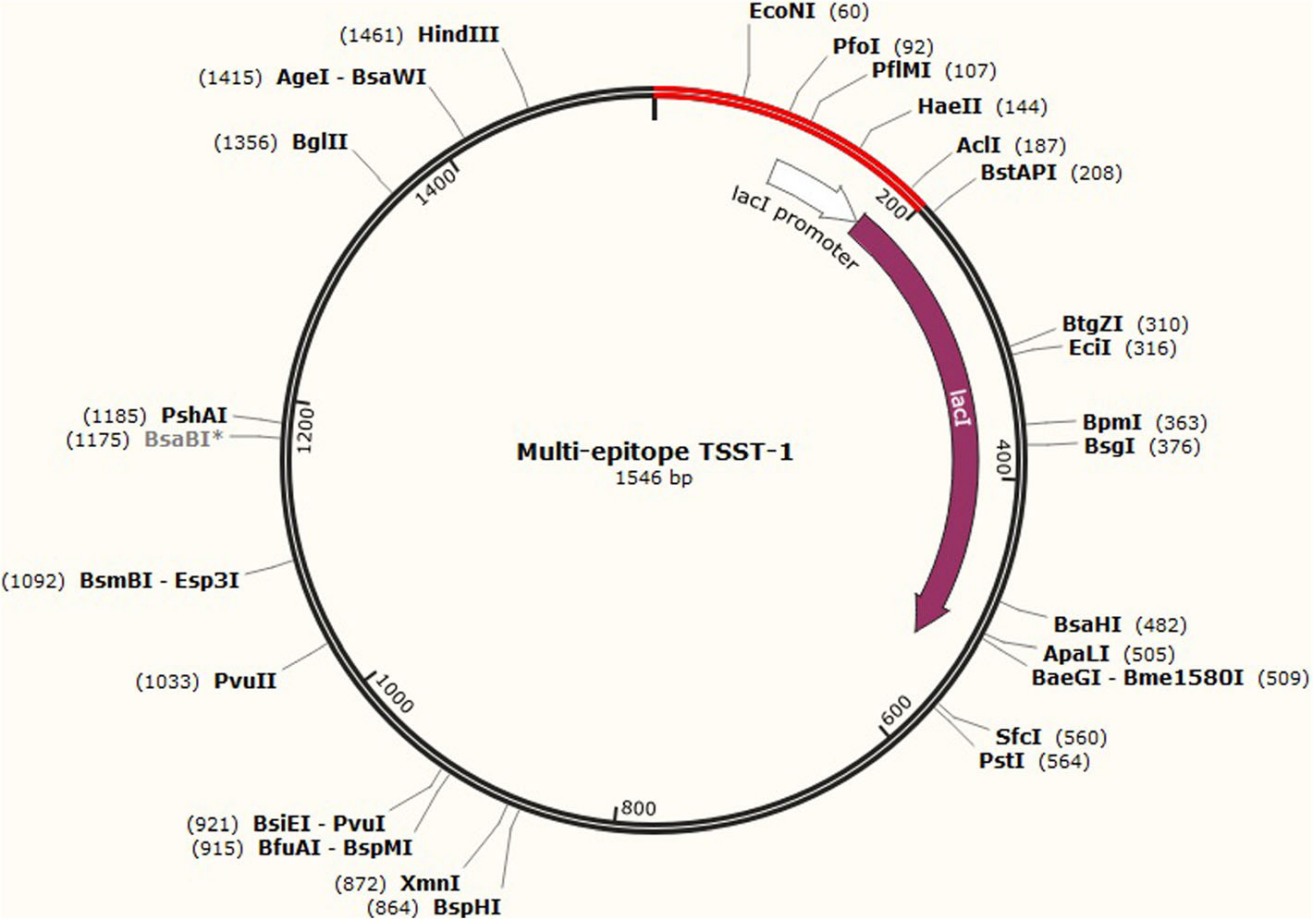
*In silico* cloning of gene coding (red color) for the vaccine construct in to pET28a (+) vector

DNA sequence that codes for the multi-epitope vaccine designed in this study was cloned *in silico* into pET-28a (+) vector between the restriction sites, *Pae*71-*Psp*XI-*Xho* and *Mlu*I. Initially, restriction sites were not present within or at the ends of the insert sequence. DNA sequence comprising sites for the restriction enzymes *Xho*I and *Mlu*I were added at the N-terminal and C-terminal ends of the vaccine construct to ensure efficient cloning without alteration in the ORF. Both insert and vector were directionally cloned *in silico* between the respective cloning sites. The final length of the pET28a (+) vector comprising vaccine insert is 1546 bp long (Fig. 9).

## Discussion

Conventional vaccines developed either through inactivation or live attenuation of the pathogen are strong enough in eliciting an immune response. But these vaccines may be allergic and toxic due to the presence of toxic and strong allergic molecules. Although recombinant vaccines (sub-unit, conjugate, and toxoid vaccines) were designed to target a specific protein or toxin, there might be chances of severe toxicity and failure of the vaccine owing to the presence of non-antigenic or allergic determinants. Multi-epitope vaccines with a reverse vaccinology approach using bioinformatics resources are now rapidly evolving. Immunoinformatics methods are targeted to cut down the efforts and cost in vaccine development and enable the design of multi-epitope vaccine in such a way that only antigenic, non-toxic, and non-allergenic determinants are included in the vaccine [39–43].

In the present study, a multi-epitope vaccine construct was designed against a pyrogenic toxin TSST-1 secreted by *Staphylococcus aureus* by employing various *in silico* tools. Epitopes were predicted for TSST-1 protein, and those epitopes which were antigenic alone were considered for the design of the vaccine. Antigenic epitopes were further assessed for their allergenicity and toxicity. Subsequently, epitopes that were antigenic, non-allergenic, and non-toxic were joined with linkers (GPGPG, AAY, and KK) and adjuvants (β-defensin and PADRE) for the construction of multi-epitope vaccine. GPGPG, AAY, and KK linkers are the most widely used linkers for the design of multi-epitope vaccines. Linkers were added between the epitopes to avoid junctional immunogenicity, thereby ensuring effective antigen presentation and enhancing sharp immunity towards the pathogen [21, 44]. B cell epitopes with greater than or equal to 10 amino acids in length alone were considered. T cell epitopes in TSST-1 protein were predicted for HLA alleles (both HLA I and II) that are most frequently occurring among the global population. B cell epitopes and HLA-II restricted T cell epitopes were joined with KK (lysine–lysine) linker. These amino acids are targets for lysosomal protease enzyme during antigen processing and presentation via MHC-II [45–47]. β-defensin and PADRE sequence were added as adjuvants at the N-terminal end to increase the immunogenicity of the vaccine. β-defensins are anti-microbial peptides involved in innate immunity and have strong affinity to TLRs (e.g., TLR3) [21]. PADRE is a 13-AA long peptide that too has a strong affinity towards TLRs and can induce CD4^+^ T helper cell-mediated immunity [21].

GPGPG linker that joins HLA-II epitopes is one such in preventing junctional immunogenicity. Apart from this, the GPGPG linker also can induce a T helper cell immune response. GPGPG linker was designed by Livingston et al. [21, 48] and considered to be a universal spacer in antigen presentation. HLA-I epitopes were joined by AAY (Ala-Ala-Tyr) linker. AAY is the site for proteasome cleavage in mammals [21, 49, 50]. Hence, these linkers were used for joining HLA-I epitopes to provide recognition sequence for proteasome cleavage and to prevent the loss of epitopes during antigen presentation. KK (Lys–Lys) linkers were used to join B cell epitopes and HLA-II epitopes. KK linker sequence is a target for lysosomal protease enzyme during the processing and presentation of epitopes via MHC-II molecules for the induction of antibodies. All the three linkers viz. KK, GPGPG, and AAY were used for joining the epitopes thus yielding a multi-epitope vaccine without junctional immunogenicity.

Multi-epitope vaccine molecule without linkers may result in a protein with a new structure that may not be able to present the targeted epitopes via MHC or HLA molecules [21, 48, 51]. Hence, linkers were added to the vaccine sequence joining various epitopes to promote the accurate presentation via MHC or HLA to prime immune responses. Both the native TSST-1 of *Staphylococcus aureus* and the multi-epitope vaccine designed in the present study were compared for their immunological properties. The multi-epitope vaccine designed in the present study is non-allergic (protective antigen = 0.9748) ensuring safety and efficacy, whereas the native TSST-1 protein (protective antigen = 0.8730) is allergic and may show adverse effects on the host.

Population coverage analysis of the multi-epitope vaccine had shown that the designed vaccine can cover and suit 83.15% of the global population. A total of 85 positive epitopes were identified in the vaccine that can induce IFN-γ production that plays an important role in the activation of macrophages and T cells [36].

The protein structure of the vaccine was predicted and validated through online resources. The vaccine was found to be highly stable through its instability index (31.93). Ramachandran plot of the vaccine showed that nearly 97% of the residues of the total protein were in a structurally favorable environment reflecting the structural quality of the protein. Interactions of the vaccine with TLR-3 were identified with molecular docking studies. The protein sequence of the vaccine was converted to DNA sequence through codon adaptation. Codon adaptation for vaccine peptide sequence was carried out to synthesize optimized DNA sequence for expression in *E coli* K12 strain. CAI value of 0.95 indicates a higher level of expression of the gene in host cells (*E coli* K12). The obtained gene insert sequence was successfully cloned *in silico* into a pET-28a (+) vector.

Immunogenicity of the vaccine was determined through immune simulations, and it was clear that the vaccine designed in the study is efficient in eliciting primary, secondary, and tertiary immune responses with a higher level of antibody production in secondary and tertiary stages of the immune response (Fig. 3a). Levels of antigen were decreased rapidly in successive intervals (Fig. 3a). Cytokine, B cell, and T cell profiles (Fig. 3b, c, d, e, f) of the vaccine indicated that the vaccine was effective and safe in conferring immunity towards the pathogen.

Although multi-epitope vaccines are highly specific, elicit targeted immune response, and avoid allergic reactions, in some cases the peptide vaccine expressing the mapped epitopes would be less immunogenic due to enzymatic degradation in the serum [52]. Certain issues concerned with such peptide-based vaccines are regarding purity and stability. The introduction of post-translational modifications such as glycosylation into the recombinant peptides is the major challenge in peptide vaccine development [53]. A present study is a computational approach towards the design and *in silico* characterization of a multi-epitope vaccine against *S. aureus* targeting the TSST-1 toxin. However, the multi-epitope vaccine designed in the present study needs to be evaluated in animal models and subsequently in clinical trials to understand and study the immune response of the multi-epitope vaccine in the host.

## Conclusion

A multi-epitope vaccine was designed in the present study through an immunoinformatics approach. Epitopes were mined and screened based on their immunological properties. Final epitopes were joined with linkers and adjuvants. Physicochemical properties and structural analyses of the vaccine were performed. The peptide sequence of the vaccine was reverse translated to nucleotide sequence through codon adaptation. The nucleotide sequence was cloned into a pET-28a (+) vector. The immunogenicity of the vaccine was studied through computer-aided immune simulations. Through immune simulation studies, it was clear that the multi-epitope vaccine could trigger both T cell and B cell responses and can elicit primary, secondary, and tertiary immune responses. *In vivo* efficacy of the vaccine construct on humans needs to be further investigated.

## Data Availability

The datasets generated during and/or analyzed during the current study are available from the corresponding author on reasonable request.

## Abbreviations

3D: Three-dimensional
AYY: Alanine, tyrosine, and tyrosine
CA: Community associated
CAI: Codon Adaptation Index
CD4^+^: Cluster of differentiation 4+
C-IMMSIM: C language version of Immune system simulator
C-score: Confidence score
CTL: Cytotoxic T lymphocytes
DNA: Deoxyribonucleic acid
*E coli* K12: *Escherichia coli* K12 strain
EAAK: Glutamic acid, alanine, alanine, lysine
GC: Guanine cytosine
GPGPG: Glycine, proline, glycine, proline, and glycine
GRAVY: Grand average of hydropathicity
HA: Hospital associated
HLA: Human leukocyte antigen
HTL: Helper T lymphocytes
IEDB: Immune epitope database
IFN-γ: Interferon γ (gamma)
IgG: Immunoglobulin G
IgM: Immunoglobulin M
IL-2: Interleukin 2
I-TASSER: Iterative Threading ASSEmbly Refinement
JCat: JAVA Codon adaptation tool
KK: Lysine and lysine
LOMETS: Local meta threading server
MHC II: Major histocompatibility complex II
MRSA: Methicillin-resistant *S. aureus*
Ni-NTA: Nickel-Nitrotriacetic acid
NK: Natural killer
ORF: Open reading frame
PADRE: Pan HLA DR-binding epitope
PDB: Protein Data Bank
PSIPRED 4.O: Predict secondary structure 4.O
RCSB: Royal chemical society of Britain
RMSD: Root mean square deviation
SA: Strong affinity
TGF-β: Transforming growth factor β (beta)
TLR-3: Toll-like receptor
TSST-1: Toxic Shock syndrome toxin 1
VISA: Vancomycin-intermediate *Staphylococcus aureus*
VRSA: Vancomycin-resistant *Staphylococcus aureus*
